# Chaperonin containing TCP1 subunit 5 is a tumor associated antigen of non-small cell lung cancer

**DOI:** 10.18632/oncotarget.19369

**Published:** 2017-07-18

**Authors:** Hongjun Gao, Min Zheng, Sijin Sun, Hongwu Wang, Zhigang Yue, Yun Zhu, Xiaochen Han, Junquan Yang, Yanqiu Zhou, Yiran Cai, Wanning Hu

**Affiliations:** ^1^ Department of Clinical Laboratory, China Meitan General Hospital, Beijing, PR China; ^2^ Department of Oncology, Tangshan People's Hospital & Tangshan Cancer Hospital, North China University of Science and Technology, Tangshan, PR China; ^3^ Department of Oncology, China Meitan General Hospital, Beijing, PR China; ^4^ Department of Pathology, Beijing Chest Hospital, Capital Medical University & Beijing Tuberculosis and Thoracic Tumor Research Institute, Beijing, PR China; ^5^ Department of Clinical Medicine, Chinese Academy of Medical Sciences & Peking Union Medical College, Beijing, PR China

**Keywords:** CCT5, non-small cell lung cancer, CEA, CYFRA 21-1

## Abstract

Novel tumor antigens and their related autoantibodies have tremendous potential for early diagnosis of non-small cell lung cancer (NSCLC). In this study, we identify antigens from NSCLC tissue and autoantibodies in sera of patients with NSCLC using a modified proteomics-based approach. We seperated and identified four NSCLC-associated proteins extracted from the cytosol in tumor tissues by mini-two-dimensional gel electrophoresis, followed by Western blot and hybridization with individual sera for confirmation of antibody binding. Of the proteins we identified, we selected 58 kDa chaperonin containing TCP1(T-Complex Protein 1) subunit 5 (CCT5) for validation. Serum levels of carcinoembryonic antigen (CEA) and cytokeratin 19 fragments (CYFRA 21-1) were measured in all serum samples with an immunoluminometric assay and a receiver operating characteristic (ROC) curve was analyzed for autoantibodies against CCT5, CEA and CYFRA 21-1. The results show that CCT5 can induce an autoantibody response in NSCLC sera and show higher expression in NSCLC tissues by immunohistochemistry and Western blot. Anti-CCT5 autoantibody was found in 51% (23/45) of patients with NSCLC, but only 2.5% (1/40) in non-tumor individual controls. A receiver operating characteristic curve constructed with a panel of autoantibodies against CCT5 (AUC=0.749), CEA (AUC=0.6758), and CYFRA 21-1(AUC=0.760) show a sensitivity of 51.1% and 97.5% specificity in discriminating NSCLC from matched controls. These results indicate the potential utility of screening autoantibodies in sera, show that CCT5 could be used as a biomarker in cancer diagnosis.

## INTRODUCTION

According to the Chinese Cancer Registry Annual Report in 2015, the incidence of lung cancer in China was predicted to be 733.3 thousand in 2015, with 610.2 thousand resulting in fatalities [1], the highest mortality rate of all cancers. The high mortality rate results both from the high incidence and the late stage of the disease at diagnosis. Lung cancer can be broadly divided into two groups---small cell lung cancer and non-small cell lung cancer (NSCLC). Nearly 85% of all lung cancer patients have NSCLC [2]. Although tremendous progress has been made in the diagnosis and treatment of NSCLC in recent years, patients with advanced disease still suffer from a 5-year survival rate lower than 20% [3]. Research shows that if a tumor is diagnosed at an early stage, the likelihood of survival dramatically improves [3]. Serum markers currently available for NSCLC diagnosis in clinical practice lack sensitivity and specificity, so the need to develop novel strategies for early diagnosis of NSCLC is urgent.

Easily accessible biomarkers for NSCLC could help to meet this need. An ideal marker would have considerably increased sensitivity and specificity and require less invasive methods for diagnosis. Autoantibodies appear to have these and other properties that make them important marker molecules with immense potential as diagnostic tools [4]. Their structures are stable and can maintain steady concentration in plasma, they are easily obtained via patients’ blood samples, and in many laboratories there are reliable quantitative methods to detect these autoantibodies. Autoantibodies were once well known for their critical role in autoimmune diseases [5], and more recent research suggests that many cancers also give rise to autoantibody production, which may be due to the presentation of tumor-associated antigens (TAAs) [6, 7]. TAAs are mutated or aberrantly expressed proteins produced during cancer onset and progression that can act as antigen to elicit an immune response [8, 9]. Chapman *et al* showed that 76% of 104 lung cancer patients presented autoantibodies, indicating that testing sera may be of great value in the early detection of lung cancer [10].

In 2001, Brichory *et al* first reported that using proteomics-based analysis can help in finding useful biomarkers in lung adenocarcinoma [11]. That experiment used a protemics based method called Proteomex (an abbreviation of proteomics and SEREX). Its synonyms, SERPA (Serological Proteomics Analysis) and SPEAR (Serological and Proteomic Evaluation of Antibody Responses), refer to the same methodology. This procedure identifies antigens directly using tumour cell lysates,which has the advantage of taking tumor-specific post-translational modification into account [12]. We have previously studied the autoantibodies in the sera of Chinese esophageal carcinoma patients using this proteomics-based analysis [13].

In this study, we examined 65 serum samples of NSCLC patients to identify autoantibodies against proteins expressed in NSCLC tissues. We found several positive spots using Western blot and four of them were confirmed by MALDI-TOF/TOF-MS. Among these identified proteins, CCT5 presents considerable sensitivity and specificity and is a promising and new diagnostic biomarker for NSCLC.

## RESULTS

### Autoantibodies against sera from NSCLC patients and identification of four reactive proteins

**Figure 1:**
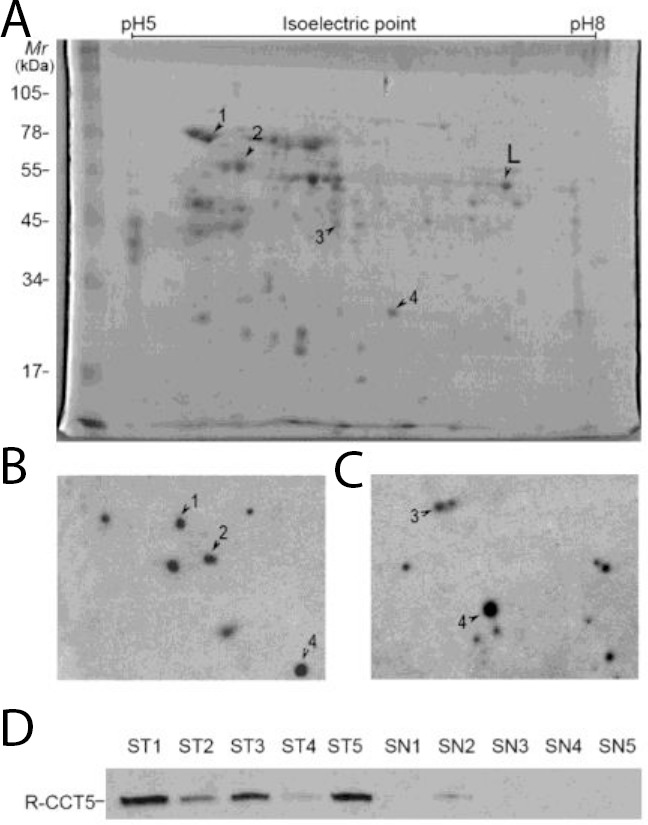
Screening and validation of autoantibodies in NSCLC **(A)** Coomassie blue staining of NSCLC cytosol proteins separated by 2-dimensional PAGE showing protein spots (1–4) recognized by sera from patients with NSCLC. **(B)** and **(C)** The map showing two representative results of 2D-Western blot performed with NSCLC patient serum against cytosol extractions of NSCLC tissues. (Most of healthy control sera was negative, data not shown). **(D)** Recombinant CCT5 (100 ng) was analyzed by Western blot and reacted with sera from 45 patients with NSCLC (ST1 to ST5) and 40 healthy subjects (SN1-SN5). Reactivity to CCT5 was shown in 23 of 45 (51%) patients with NSCLC and in 1 of 40 (2.5%) of healthy subjects. Representative results shown. L: land marker in mapping.

**Figure 2:**
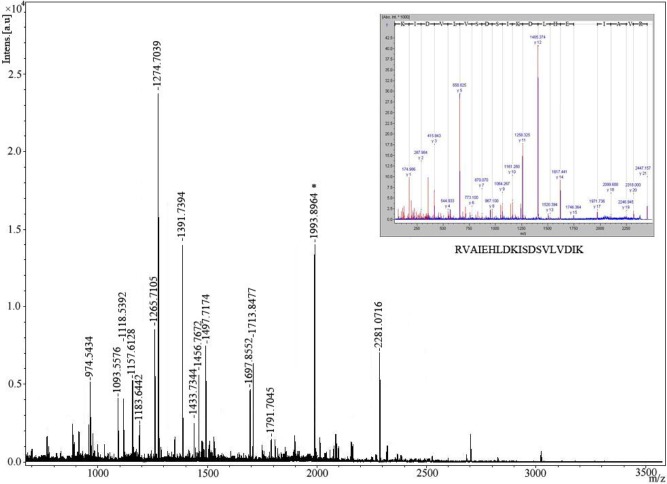
Identification of protein spot 2 recognized by sera from patients with NSCLC by mass spectrometry The identification of CCT5 was performed by MALDI-TOF MS after trypsin digestion of the protein. Inset: The tandem mass (MALDI-TOF/TOF) spectrum of one peptide, M/Z=1993.8964 (starred).

NSCLC tissue cytosol proteins were separated by two dimensional SDS-PAGE (Figure 1A) and then transferred onto PVDF membranes. Sera from 20 patients with NSCLC and from 20 Non-tumor individuals were screened individually for the presence of autoantibodies. The results indicated that 80% (16/20) of NSCLC patient sera contained autoantibodies recognizing tumor cytosol proteins, while only 2 out of 20 (10%) non-tumor individuals showed positive reactivity. A total of four protein spots with different expression levels were found on 2-DE and were eventually identified by MALDI-TOF/TOF MS (Figure 1B and 1C). The acquired spectra were processed and searched against a Mascot Search engine based on the entire NCBIn and SwissProt protein databases (Figure 2). Spots 1-4 were identified as chaperonin containing TCP1 subunit 5 (CCT5), heat shock 70kDa protein (HSP70) 8 isoform 1, eukaryotic translation elongation factor 1 (eEF1) gamma, and phosphoglycerate mutase 1 (PM1) (Table 1). The protein mass function (PMF) and identified peptide sequence of MALDI-TOF/TOF for CCT5 are shown in Figure 2.

**Table 1:** Characteristics of NSCLC serum specimens

	TAAs primary screening			Autoantibody against CCT-5 validation		
	No.	Age range (years)	Age mean (years)	No.	Age range (years)	Age mean (years)
Gender						
Male	14	42-83	62.5	29	43-88	63.7
Female	6	43-81	60.2	16	40-82	62.9
Stage						
I	0			10	59-65	62
IIa	2	43-83	60.5	15	40-88	61.2
IIb	3	44-70	64.2	18	43-82	65.9
III	15	42-81	58.8	2	41-73	57
IV	0			0		

### Diagnostic evaluation of autoantibodies against CCT5 in sera of NSCLC patients

**Figure 3:**
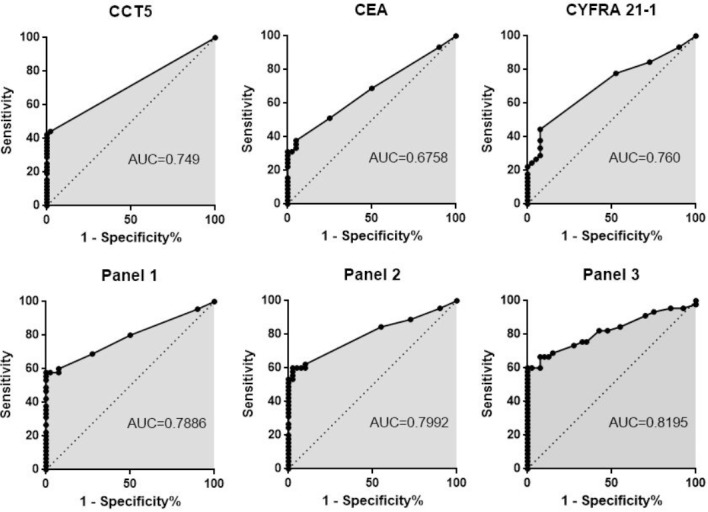
ROC curves for CEA, CYFRA21-1, autoantibody against CCT5 and their panels of two or three of the three proteins combined ROC curves for 45 patients with NSCLC and 40 healthy subjects. The AUC is indicated. Panel 1: autoantibody against CCT5 combined with CEA, Panel 2: autoantibody against CCT5 combined with CYFRA 21-1, Panel 3: autoantibody against CCT5 combined with CEA and CYFRA 21-1.

To evaluate CCT5 as a diagnostic antigen, commercial recombinant CCT5 proteins were separated by SDS-PAGE and then transferred to a PVDF membrane for Western blot. Western blots against recombinant CCT5 showed reactivity in 51% (23/45) of sera from patients with NSCLC but only 2.5% (1/40) of non-tumor individuals (Figure 1D). Out of 45 serum samples from patients with NSCLC, 40% (18/45) had high CEA levels and 49% (22/45) had high CYFRA 21-1 levels. Out of 40 serum samples from non-tumor individuals, 5% (2/40) had high CEA levels and 2.5% (1/40) had high CYFRA 21-1 levels. The sensitivity of the antibody against CCT5 for identifying stage I NSCLC was 20% (2/10). The sensitivities of CEA and CYFRA 21-1 for identifying stage I NSCLC were 10% (1/10) and 20% (2/10), respectively. The sensitivity of the autoantibody against CCT5 was not significantly different from that of CYFRA 21-1, however, the sensitivity of the autoantibody against CCT5 was significantly higher than that of CEA for identifying stage I NSCLC (*P* <0.05). When the data for autoantibody against CCT5, CEA, and CYFRA 21-1 were combined into a panel and the samples positive for NSCLC were pooled, the sensitivity was 40% (4/10) for identifying stage I NSCLC, which was significant higher than the sensitivity of any individual protein (*P*<0.05). Autoantibody reactivity against CCT5, CEA, and CYFRA 21-1 was also evaluated by ROC analysis. The areas under the curve (AUC) were 0.749, 0.6758, and 0.760 for autoantibodies against CCT5, CEA, and CYFRA 21-1, respectively (Figure 3). Combining data for autoantibody against CCT5 and CEA as panel1, autoantibody against CCT5 and CYFRA 21-1 as panel2, and autoantibody against CCT5, CEA and CYFRA 21-1 as panel3, then summing the ranks for each sample yielded an AUC of 0.7886, 0.7992 and 0.8195, respectively.

### Immunohistochemical analysis of CCT5

**Figure 4:**
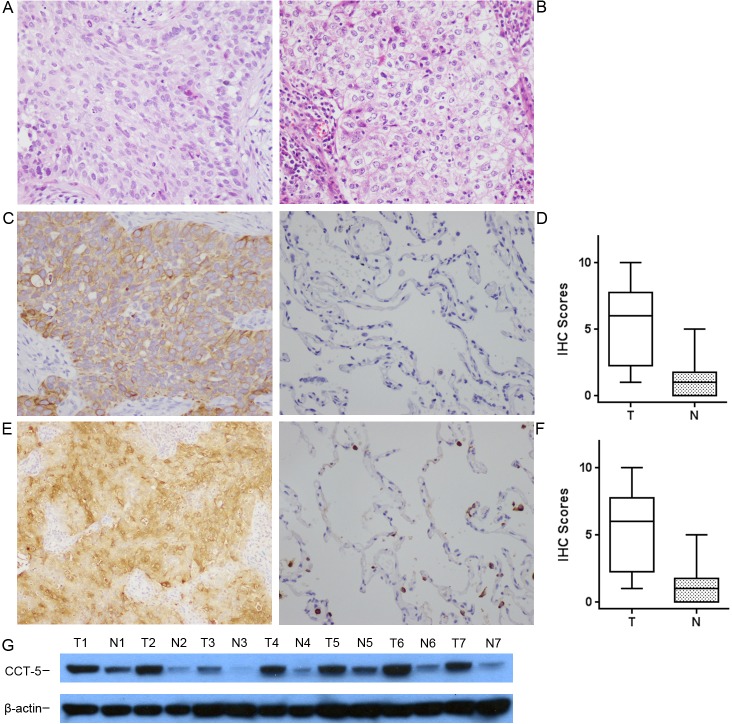
The expression of CCT5 in cancer and non-cancer epithelium were assessed by immunohistochemistry with monoclonal antibodies **(A)** Squamous cell lung carcinoma and **(B)** lung adenocarcinoma were stained with hematoxylin and eosin. Expression of CCT5 was observed mainly in the cytoplasm of squamous cell lung carcinoma **(C)** and lung adenocarcinoma **(E)**, but was not detected in normal epithelium **(D)**, or was expressed much more weakly **(F)**. Expression of CCT5 was confirmed by Western blot **(G)** in paired tumor and non-tumor tissues. The representative results were showed that the expression of CCT5 was significantly higher in tumor tissues (T1-T7) than that in normal lung tissues (N1-N7). The right box plots represent the statistical distribution of IHC scores for CCT5 in squamous cell lung carcinoma (top, *P*<0.01) and in lung adenocarcinoma (bottom, *P*<0.01). All images are presented at 200x magnification.

The expression of CCT5 in tumor tissues from 20 patients with squamous cell lung carcinoma and 20 patients with lung adenocarcinoma and their adjacent non-tumor tissues were assessed immunohistochemically and by western blot using goat anti-mouse monoclonal antibody. In squamous cell lung carcinoma, higher expression of CCT5 tended to be localized only in the cytoplasm, and the adjacent non-tumor tissues showed lower or non-expression (Figure 4C and 4D). The expression pattern of CCT5 in lung adenocarcinoma was the same as that in squamous cell lung carcinoma (Figure 4E and 4F). The expression of CCT5 in tumors was significantly higher than that in the adjacent non-tumor tissue, which was confirmed by Western blot (Figure 4G). The rates of positive expression of CCT5 in lung squamous cell carcinoma and adenocarcinoma were 60%(12/20) and 65%(13/20), respectively.

## DISCUSSION

A lot of progress has been made in cancer immunology in the past decade [14]. The discovery of novel tumor antigens led to early diagnosis and targeted molecular therapy for certain tumors [15, 16]. Although the specific mechanism by which a tumor becomes immunogenic and acquires specific antigens remains unclear, it is hypothesized that increasing protein expression and posttranscriptional modifications in tumor tissue could enhance immunogenicity [17, 18]. Therefore, serum samples from tumor patients could be of great importance in detecting the presence of tumor antigens and corresponding antibodies. These antibodies indicate that a humoral immune response has been induced, which implies that the immune system may play a role in cancer surveillance. This theory is supported by Burnet *et al*. who found that tumors are rejected in syngeneic hosts while normal tissues are not [19]. The humoral immune response against cancer reflects tumor antigen profiles and can be used to detect early forms of cancer.

Serological Proteomics Analysis (SERPA) is a classical method combining two-dimensional electrophoresis (2-DE) and MALDI-TOF/TOF MS to separate and identify proteins [20, 21]. Unlike the former method (SEREX) which constructs a cDNA library from tumor tissue to screen for tumor-associated antigens (TAA) [22–24], SERPA screens for TAA directly from tumor cell lysates and thus is closer to the natural state of the protein. The advantage of this procedure is that it takes PTM (post-translational modification) into consideration [12, 25, 26], which allows the identification of antigens that are immunogenic due to PTM. Our previous study showed that deglycosylated TIM (triosephosphate isomerase) cannot be recognized by autoantibodies [13], thus PTM is crucial for antigen-antibody reaction in at least some cases. Several TAAs have been identified using SERPA and demonstrate the reliability of this strategy in identifying novel tumor antigens [13, 27–32]. In addition, SERPA includes normal tissue as background in the screening procedure, conferring tumor specificity of antigens recognized by patient sera. SERPA does have a relatively low coverage of potential antigens due to limited capability of 2-DE to separate proteins [21], which will be addressed in future work.

In the present study we identified four candidate tumor-associated antigens, including chaperonin containing TCP1 subunit 5 (CCT5), heat shock 70 kDa protein 8 isoform 1 (HSP70), eukaryotic translation elongation factor 1 gamma (eEF1), and phosphoglycerate mutase 1 (PGM1). PM1 and HSP70 have already been investigated extensively by other groups and were not studied further in these experiments [33–36]. The eukaryotic translation elongation factor 1 gamma has also been previously identified as a potential diagnostic and prognostic factor for NSCLC [37, 38]. Therefore our study primarily focused on CCT5. Chaperonins are a family of chaperone proteins that guide misfolded and naive proteins to their native states in an ATP-dependent manner [39], an essential process in sustaining a living cell. There are two main groups of chaperonins. Group I is found mainly in prokaryotes and inside organelles (mitochondria, for example) while group II is found in the archaeal and eukaryotic cytosol. The eukaryotic group II chaperonin, formally named TCP-1 ring complex (TRiC), consists of two identical rings, with each ring containing eight different chaperonins with TCP-1 (CCT) subunits [40]. There are different arrangements and ratios of CCT subunits in different TRiCs, and this complex may have different substrates and varying affinity with ATP [41]. Although it is known that CCT5 has a high ATP-binding affinity and inhibits mutant protein aggregation [41, 42], the specific role of the TRiC CCT5 complex is still unclear. Several studies have shown that TRiCs are involved in oncogenesis and tumor progression through modulation of cancer related proteins. The known substrates of TRiCs include p53 [43] and STAT3 [44] as well as CDC20 [45]. Recent studies also suggest that TRiC is involved in leukemogenic processes through interactions between the DNA-binding domains of oncoprotein [46]. Sergeeva *et al*. successfully expressed human CCT5 in *Escherichia coli* and surprisingly found that this subunit can exhibit chaperonin activities when organized into homo-oligomeric rings [47]. Other research suggests that tumor suppressor proteins (TSPs) are candidate substrates for TRiC containing CCT1 and CCT7 [48]. Together these findings suggest that expression of CCT5 in tumor tissue may be involved in tumor progression by inhibiting the function of TSPs, due to inactivated chaperonin.

We have investigated the presence of antibodies against CCT5 in sera from NSCLC for the first time. To evaluate its biomarker potential, we conducted western blot analysis in sera from tumor and non-tumor patients and found that antibody response was detected in 51% (23/45) of NSCLC and in only 2.5% (1/40) non-tumor individuals. We also confirmed that antibody was only present in patients with advanced-stage disease, as stage 1 patients had a reactivity rate of 20% (2/10) as well. When a combination of CCT5, CYFRA 21-1 and CEA was used to detecting early stage disease, the response was found in 40% (4/10) of stage I patients, which was significant higher than the response detected from any protein alone (*P*<0.05). Our results indicate that CCT5 is a potential tumor marker and may be useful in the diagnosis of NSCLC in an early stage. Using immunohistochemical methods, we showed that higher expression of CCT5 tended to be localized only in the cytoplasm of tumor cells rather than in adjacent non-tumor tissues. These findings again demonstrate that CCT5 is involved in oncogenesis, though the specific contributions of CCT5 to tumor formation are not yet known. Further studies in structural biology and biochemistry may provide insight into these questions.

As our sample size was small, large-scale clinical research is needed to further validate our conclusions. It was also not possible for us to evaluate the diagnostic efficacy of CCT5 according to the TNM classification system due to the small sample size. Smoking and COPD were confounding factors in our study, and future studies should include more specific inclusion criteria to avoid these issues. Ideal diagnostic processes would incorporate sensitivity and specificity signals with clinical presentation and imaging findings to develop a multiple factor lung cancer screening model which takes biological indicators and patient characteristics into consideration. In addition, more quantitative studies are needed to determine the relationship between the level of CCT5 expression and TNM classification and whether CCT5 and other CCT subunits can be a prognosis factor.

## MATERIALS AND METHODS

### Serum samples

The sera from 65 (20+45) newly diagnosed subjects with NSCLC were obtained from China Meitan General Hospital, Tangshan People's Hospital & Tangshan Cancer Hospital, Age matched control sera from 60 (20+40) individuals who had no obvious evidence of malignancy and chronic obstructive pulmonary disease (COPD) were obtained from China Meitan General Hospital. Some common lung diseases such as inflammation, infection, trauma, and benign pulmonary disease were exclusive in mathced control. Neither group was restricted to non-smoking subjects. The serum samples were randomly divided into two sets to yield one primary screening set consisting of 20 patients with pathological evidence of NSCLC and 20 matched controls, and a second independent set consisting of 45 serum samples from patients with NSCLC patients and 40 matched controls. The basic information of patients was showed in Table 2. This study was approved by the ethics committees of both hospitals.

**Table 2:** MS identification of the TAAs recognized by autoantibodies in NSCLC serum

Spot no.^a^	Pep. match	Accession no.^b^	Description	Score	Molecular mass(Da)		pI	
					Theor.^c^	Observ^d^.	Theor.^e^	Observ.^f^
1	19/27	gi|62897129	heat shock 70kDa protein 8 isoform 1	199	70855	70000	5.37	5.50
2	17/37	gi|24307939	chaperonin containing TCP1, subunit 5	250	59633	58000	5.45	5.75
3	11/32	gi|4503481	eukaryotic translation elongation factor 1 gamma	87	50087	50000	6.25	6.33
4	24/41	gi|49456447	phosphoglycerate mutase 1	418	28817	25000	6.67	7.2

a. Protein spots described in Figure 1.

b. NCBI database accession number

c. Theoretical molecular mass (Da)

d. Observed molecular mass (Da)

e. Theoretical isoelectric point

f. Observed isoelectric point

### Cancer tissue and protein extracts

The cancer tissues from 20 patients with squamous cell lung carcinoma and 20 patients with lung adenocarcinoma were obtained from Beijing Chest Hospital, Capital Medical University and Beijing Tuberculosis and Thoracic Tumor Research Institute. Five samples of squamous cell lung carcinoma and five samples of adenocarcinoma were rinsed twice with PBS buffer (137 mM NaCl; 2.7 mM KCl; 10 mM Na_2_HPO_4_; 2 mM KH_2_PO_4_), respectively. Subcellular proteins were extracted and pooled all them following the ProteoExtract Subcellular Proteome Extraction Kit (Merck, Darmstadt, Germany). The resulting mixture of proteins was used for a two-dimensional Western blot. This experiment was approved by the ethics committee of Beijing Chest Hospital.

### Two-dimensional western blot

To concentrate proteins and remove impurities such as salts or detergents that may interfere with 2-D SDS-PAGE separation, the extracts were precipitated with the ProteoExtract protein precipitation kit (Calbiochem Darmstadt, Germany). The pure cytosol proteins were solubilized in sample rehydration buffer (8 M urea, 2% CHAPS, 0.5% ZOOM Carrier Ampholytes). 2-DE was carried out in a ZOOM IPGRunner System (Invitrogen, Carlsbad, CA). Immobilized pH gradient (IPG) strips 7 cm in length with pH ranges of 5–8 were rehydrated overnight in 155 μL of the sample rehydration buffer containing traces of bromophenol blue, 20 mM DTT, and 100 μg protein. The first dimension was performed using a multistep protocol (25 min at 200 V, 15 min at 450 V, 15 min at 750 V, and 100 min at 2,000 V). At the end of isoelectric focusing, each strip was equilibrated in two steps of 15 min in 5 mL of Laemmlisample buffer supplemented with 10 mg/mL DTT instead of β-mercaptoethanol, and 40 mg/mL iodoacetamide, respectively. The second-dimension SDS-PAGE and Western blot were performed as described below. The preparative gel was stained with Coomassie blue in accordance with the manufacturer's instructions for antigen identification.

### In-gel digestion

The proteins of interest were excised from the Coomassie blue-stained preparative gel and then washed with high performance liquid chromatograph (HPLC) grade water, destained with acetonitrile for 15 min to remove Coomassie blue staining, and dried in a vacuum centrifuge (Eppendorf, Westbury, NY) as described previously [49]. The digestion was performed by the addition of 12.5 ng/μL sequencing-grade trypsin (Promega, Madison, WI) in 40 mM ammonium bicarbonate containing 4 mM CaCl_2_. Following the enzymatic digestion overnight at 37°C, the peptides were extracted with 25 mM ammonium bicarbonate in 50% acetonitrile, followed by 2.5% formic acid in 50% acetonitrile solution. After the removal of acetonitrile in a vacuum centrifuge, the sample was desalted by C18 bead ziptips (Applied Biosystems, Framingham, MA) and dried by a vacuum centrifuge before mass spectrometry analysis.

### Protein identification

Protein identification was repeated at least twice using spots from different gels. The obtained peptide mass fingerprint (PMF) was used to search through the Swiss-Prot and National Center for Biotechnology Information nonredundant (NCBInr) databases by the Mascot search engine (www.matrixscience.co.uk). Protein identification was reconfirmed by an MALDI-TOF/TOF MS approach. The database search was finished with the Mascot search engine (www.matrixscience.co.uk) using a Mascot MS/MS ion search. In addition, the amino acid sequences of the peptides were deduced with the peptide sequencing program MasSeq.

### Immunohistochemical analysis

The expression of CCT5 in 40 matched cancer tissues (20 squamous cell lung carcinoma and 20 lung adenocarcinoma) was assessed immunohistochemically using mouse anti-CCT5 monoclonal antibody. Tissue slices were then incubated overnight at 4°C with antibody (Novus Biologicals, CO, USA) or control IgG (5 μg/mL) at a 1:200 dilution in a humidified chamber. After washing, biotinylated anti-mouse immunoglobulin (Maxim Biotech., China) was applied at a 1:100 dilution for 30 minutes at room temperature, followed by incubation with streptavidin-conjugated horseradish peroxidase. Finally, 3, 30-diaminobenzidine was used for color development and hematoxylin was used for counterstaining. A score of 4-12 was defined as “positive expression” and a score of 0-3 as “negative”. The statistical distribution was described by the box plot of the immunohistochemistry (IHC) scores for CCT5 in squamous cell lung carcinoma and CCT5 in lung adenocarcinoma.

### Western blot

The cytosol proteins of cancer tissues and adjacent non-tumor lung tissues were mixed with sodium dodecyl sulfate-polyacrylamide gel electrophoresis (SDS-PAGE) sample lysis buffer (2% SDS; 20% glycerol; 50mM Tris-HCl, pH 6.8). Thirty micrograms of protein from each matched tumor and non-tumor sample was resolved in continuous 12% Tris-HCl SDS-PAGE gel. Proteins were transferred to a Polyvinylidene Fluoride (PVDF) Membrane (0.45 μm; Bio-Rad, Hercules, CA, USA) and was blocked for 15 min at 25°C in TBS-T (20 mM Tris-HCl, pH 7.5; 50 mM NaCl; 0.05% Tween-20) buffer containing 5% skimmed milk. The PVDF membrane was incubated for 120 minutes at room temperature with a 1:2000 dilution antibody against CCT5 (Novus Biologicals, CO, USA). As secondary antibody, horseradish peroxidase–conjugated goat anti–mouse IgG (Jackson, PA, USA) was applied at a 1:5,000 dilution. Immunoreactive bands were detected with an ECL kit (YuanPingHao Bio, Beijing, China) according to the manufacturer's instructions and followed by autoradiography. Western blot films were scanned in transmittance mode in a ScanMaker S430 scanner using 300 dpi.

### Immunoluminometric assay for CEA and CYFRA 21-1

Serum carcinoembryonic antigen (CEA) and cytokeratin 19 fragments (CYFRA 21-1) levels were measured with an immunoluminometric assay on a random-access analyzer (Architect i2000, Abbott Diagnostics Division). The recommended cut-off limits for CEA and CYFRA 21-1 were 5 ng/mL and 3.3 ng/mL, respectively. Intra-assay analytical variation was minimized by using the same lot of reagent on the same day. Two quality control samples of two different concentrations of the analytes were included in each assay run, and Westgard Multi-rules were used to accept or reject runs [30]. A high CEA level was defined as exceeding 5 ng/mL, and a high CYFRA 21-1 level was defined as exceeding 3.3 ng/mL, according to the guidelines defined by the manufacturer of the test kit.

### Statistical analysis

We used the χ^2^ or Fisher's exact test to quantify the sensitivity differences of the preoperative serum autoantibody against CCT5, CEA,and CYFRA 21-1 levels. Statistical significance was set at *P* < 0.05. The diagnostic significance of the autoantibody against CCT5, CEA, and CYFRA 21-1 was analyzed with a receiver operating characteristic curve (ROC) analysis.
